# Ernst Haeckel, Nikolai Miklucho-Maclay and the racial controversy over the Papuans

**DOI:** 10.1186/s12983-020-00358-w

**Published:** 2020-05-25

**Authors:** Georgy S. Levit, Uwe Hossfeld

**Affiliations:** Friedrich-Schiller-University Jena, AG Biologiedidaktik, Am Steiger 3 (Bienenhaus), 07743 Jena, Germany

**Keywords:** History of biology, Ernst Haeckel, Nikolai Miklucho-Maclay, Racial theories, Darwinian anthropology, the Papuans, Stationary field studies

## Abstract

**Background:**

The “German Darwin” Ernst Haeckel (1834–1919) was a key figure during the first “Darwinian revolution“, a time when the foundations of the modern evolutionary theory were laid. It was Haeckel, who crucially contributed to the visualization of the Darwinian theory by designing “genealogical-trees” illustrating the evolution of various species, including humans. Although the idea of explaining human evolution by natural selection belongs to Darwin, Haeckel was the first who attempted to create a new exact anthropology based on the Darwinian method.

**Discussion:**

Trying to immediately reconstruct human evolution proceeding from the description of modern populations led Haeckel to the views which, from the contemporary perspective, are definitely racist. Haeckel created racial anthropology intending to prove human origins from a lower organism, but without the intention of establishing a discriminatory racial praxis. Although hierarchical in its outcome, the Haeckelian method did not presuppose the necessity of a racial hierarchy of currently living humans. It is crucial to grasp in what sense Haeckel’s theoretical explorations in human evolution were racist, and in what sense they were not. Our argument flows as follows. One of Haeckel’s pupils was the Russian ethnographer, anthropologist and zoologist Nikolai Nikolajewitsch Miklucho-Maclay (1846–1888). Maclay and Haeckel worked closely together for several years; they traveled jointly and Maclay had enough time to learn the major methodological principles of Haeckel’s research. Yet in contrast to Haeckel, Maclay is regarded as one of the first scientific anti-racists, who came to anti-racist views using empirical field studies in Papua-New Guinea.

**Conclusions:**

We claim that while conducting these studies Maclay applied scientific principles to a significant extent acquired from Haeckel. The paper contributes to the view that Haeckel’s theoretical racism did not follow the Darwinian method he used.

## Background

*“I believe, for instance, that Mr. Haeckel is the worst and the most harmful of all I know”*^1^.

*“You were the first, who, from your own experience, clearly demonstrated that humans are everywhere humans, that they are friendly, social creatures”*^2^.

**Fig. 1:**
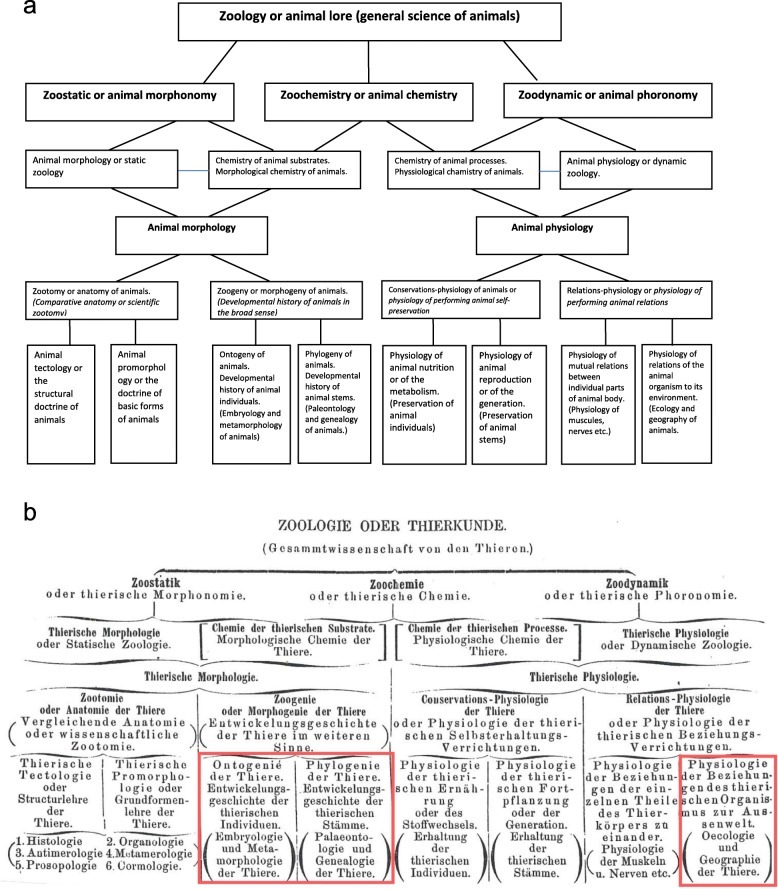
**a** and **b** Diagram - Haeckel’s overview of zoology, where he introduced his famous concepts of ontogeny, phylogeny and ecology. The scheme incorporates both the ‘static’, and ‘dynamic’ aspects of animal life, where ‘static’ is used to describe structures and forms (morphology), and ‘dynamic’ deals with a state of change. Translated by Levit and Hossfeld [40] and reproduced by the authors with slight changes from the original scheme in: Haeckel 1866, vol. 1, p. 238

Ernst Haeckel defended and developed the Darwinian theory with a passion and energy like nobody else on the continent [18, 24, 35, 37, 48, 54, 57]. Yet, he was influential not only in Germany but in non-German speaking countries as well [56, 58]. Haeckel created a conceptual framework within which the majority of Darwinians worldwide worked over decades. Contemporary evolutionary theory is unthinkable without notions coined by Haeckel such as “phylogeny”, “ontogeny”, “phylum”, “phylogenetic tree”, “gastraea theory” or “ecology” [22, 23, 25, 38, 40, 47, 67, 70] (Fig. 1).

**Fig. 2:**
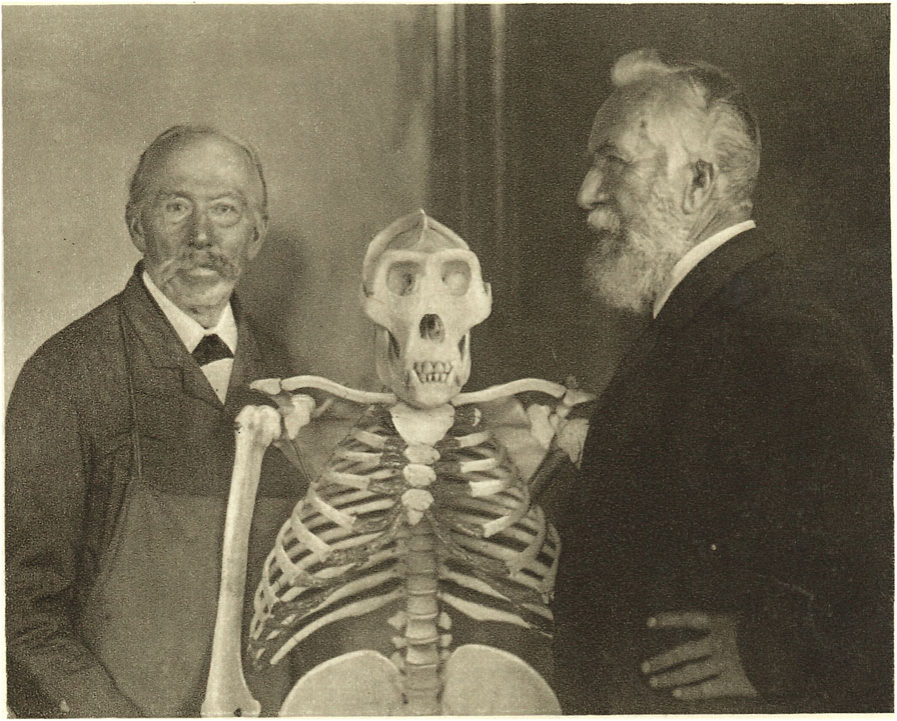
Ernst Haeckel and his “Famulus” Pohle with a Gorilla gigas skeleton in 1898 in Jena (Ernst Haeckel im Bilde. Hrsg. Walther Haeckel. Berlin: Georg Reimer, 1914)

Moreover, his theories were encouraged and admired by Darwin himself. Ernst Haeckel is, without doubt, a crucial figure in the growth of Darwinian thought in the nineteenth century. As Robert Richards emphasized: “More people at the turn of the century learned of evolutionary theory from his pen than from any other source, including Darwin’s own writings” [56]. Haeckelian (biological) anthropology was part of his Darwinian project and played an especially important role because of its immediate political concerns for a broad audience [19, 49] (Fig. 2).

Our intention in the present paper is to demonstrate that Haeckel created an anthropology that looks extremely racist from the contemporary viewpoint. It would even look racist from the viewpoint of some of his contemporaries and even his pre-Darwinian predecessors in biological anthropology. Already Johann Friedrich Blumenbach (1752–1840) stressed the essential sameness of the human species [59]. Alexander von Humboldt (1769–1859) spoke in his famous *Cosmos* about “the unity of human race” and provided several arguments in favour of this view including the form of the skull [28].

Haeckel proceeded from the assumption that humanity consists of various human species being on various stages of phylogenetic evolution. Some of these species were closer to a hypothetical pre-human ancestor, whereas some of them were more evolutionarily advanced. The division into higher and lower races is at the core of any discriminatory racism. We claim, however, that Haeckel was a Darwinian thinker, and that his method was not predestined to bring about discriminatory racial schemes. His racism followed from the lack of empirical evidence and from the temptation to explain human evolution prematurely, instead of waiting for reliable data. Applying the same method, one could have come to principally different results with access to better field studies information.

**Fig. 3:**
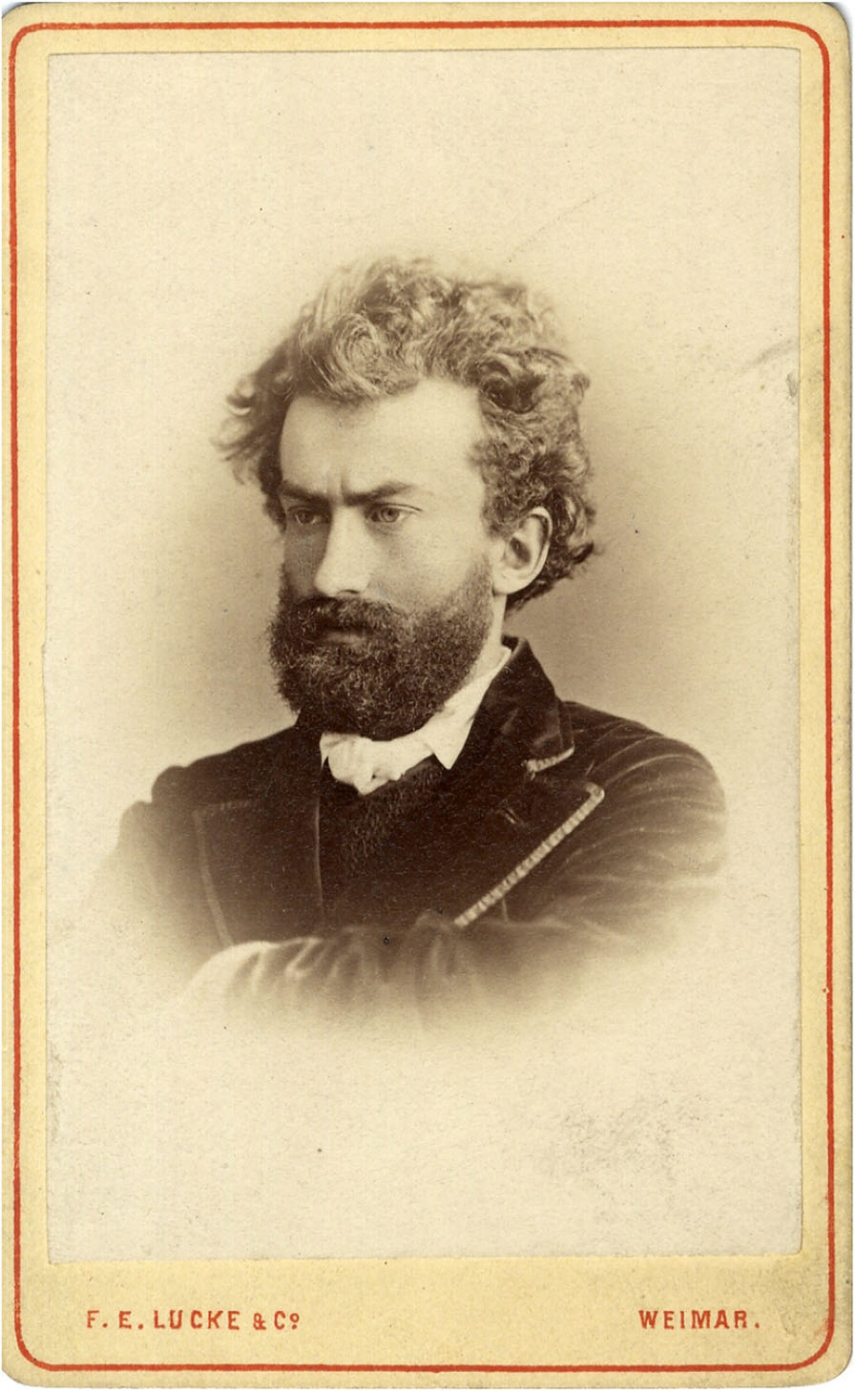
Portrait of Maclay in Weimar (Germany). Photo with his signature (on the backside) as a present to Petr Semenov, 1870. RGO, File 6, List 3, № 20

The stationary, long-term anthropological and ethnographic studies were conducted by Haeckel’s immediate pupil, the Russian traveler Nikolai Miklucho-Maclay (Miklucho-Maclay’s originally Russian name allows various spelling in other languages such as Mikloucho, Miklouho, Mikluho, Mikluha, etc. We use here the spelling he used in initial German publications). Maclay remains relatively unknown in contemporary English-speaking evolutionary anthropology and history of biosciences. English language publications on Maclay “are mostly translations of Miklucho-Maclay’s archival texts from Russian, with a scholarly commentary” [64]. At the same time, Maclay’s significance cannot be overestimated. He was arguably one of the first scientists who empirically demonstrated the unity of humanity by disproving the dominating racial anthropologies of Haeckel, Fritz Müller (1822–1897) and other influential theorists. This is at odds with his humble status in current historiography. Maclay was not only a descriptive scientist. He tried to convert his discoveries into political actions struggling against racial prejudices of his time, claiming equal rights of indigenous populations of Melanesia. The intriguing detail of this story is that Maclay came to his anti-Haeckelian and anti-racist concept by applying essentially the Haeckelian method (Fig. 3).

## Discussion

Here we outline Haeckel’s theory of human species and specify the place of the Papuans in it. We then demonstrate how Maclay jeopardized the whole Haeckel’s racial anthropology by empirically demonstrating that the Papuans were not an inferior human species and thus contributed the concept of the unity of humanity.

### Haeckel’s racial anthropology

When Darwin introduced his theory of evolution, one of the sharpest controversies arose around the origin of man. Haeckel was convinced early on that Darwinian principles are applicable to human evolution. Having only two fossil remnants (*Neanderthalensis* and *Pithecanthropus*) at his disposal, Haeckel tried to reconstruct human phylogeny as well as global migration of ancient humans [40]. Darwin reacted favorably to Haeckel’s anthropological publications. In the introduction to *The Descent of Man* [5] he wrote referring to Haeckel’s *Natürliche Schöpfungsgeschichte* [*The History of Creation*] [13]: “Almost all the conclusions at which I have arrived I find confirmed by this naturalist, whose knowledge on many points is much fuller than mine”. Praising his contemporaries and predecessors, who shared his view that man descended from a hypothetical extinct lower form, Darwin enumerated “several eminent naturalists and philosophers”, but emphasized a superior role of Haeckel, by saying that this conclusion was maintained “especially by Häckel” [5]. Darwin even admitted that if Haeckel’s work had appeared before his *The Descent of Man* had been written, he “should probably never have completed it” [55].

**Fig. 4:**
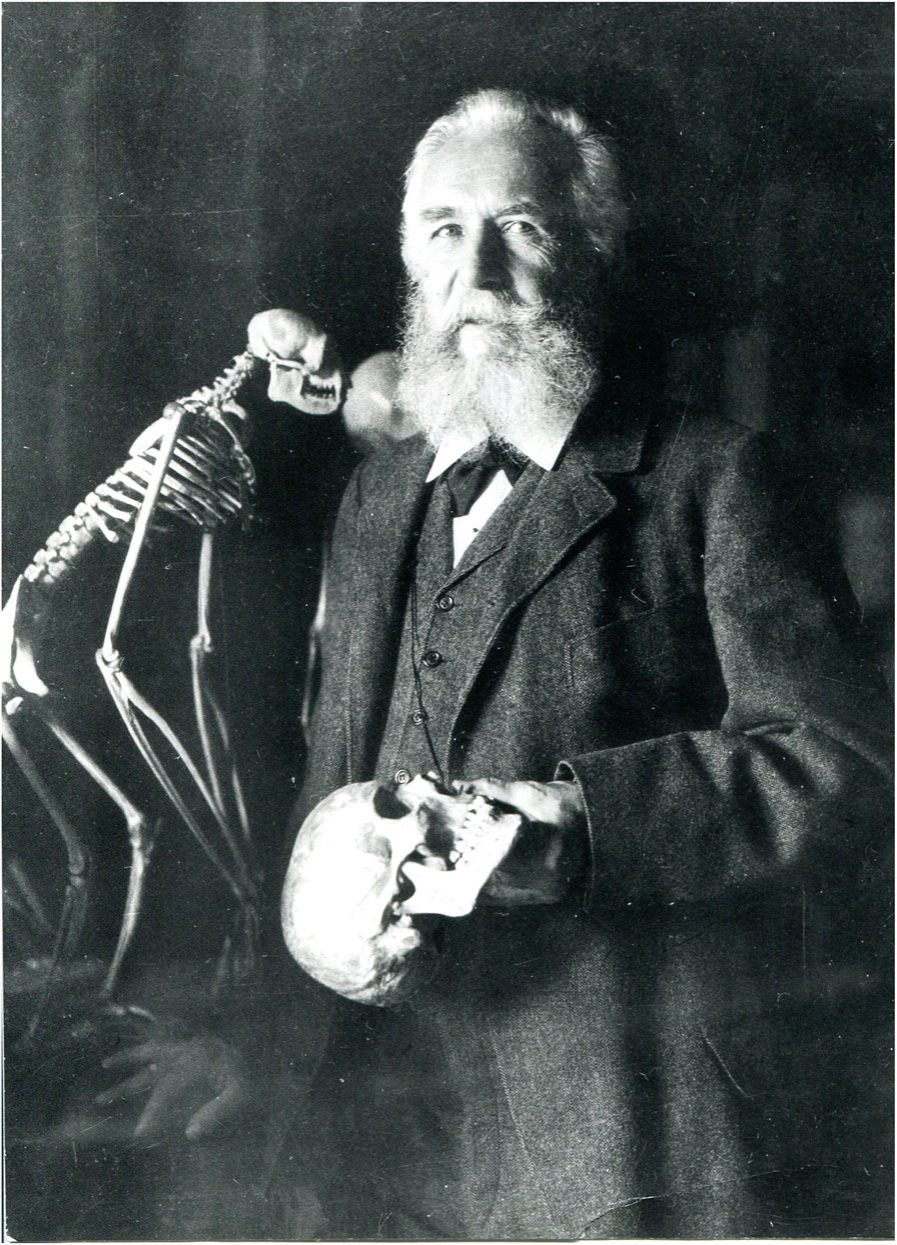
Haeckel in 1898 with the skull (Private Archive of Uwe Hossfeld)

Haeckel was occupied with human phylogeny for 45 years, beginning with the *Stettiner Vortrag* (*Speech in the city of Stettin*) in 1863 and ending with *Unsere Ahnenreihe* (Our ancestors) (*Progonotaxis Hominis*) in 1908 [19, 40] (Fig. 4). In contrast to Darwin, who merely postulated the descent of man from an ape-like ancestor, Haeckel tried to reconstruct and visualize the exact pathways of human origin. For him, it was a task of a very special mission: “Of all the individual questions answered by the Theory of Descent, of all the special inferences drawn from it, there is none of such importance as the application of this doctrine to Man himself” [15]. But these efforts made him into a highly controversial figure. Pushing forward a hypothesis that is widely accepted today, that several human species co-existed on Earth, Haeckel suggested that the alternative human species may still exist, and proposed his famous “phylogenetic trees” to capture this idea.

It was these trees that made Haeckel vulnerable to accusations of racism. He was even regarded as complicit in Nazi biology later on, but, in fact, despite isolated attempts to use his fame to support Nazi ideology, his doctrine was seen by the Nazis as unsuitable for their purposes [19, 55, 62]. The same would hold for Haeckel’s closest followers after his death, even those seeking to render Haeckel attractive to Nazi ideology [62]. Although there are several explanations for it possible, his “phylogenetic trees” were hardly suitable for actual racial discrimination because of their speculative nature whose provisional character Haeckel himself many times emphasized [22, 23, 40]. But it was also because his racial hierarchy did not correspond to conventional racism, since he placed, for example, Jews and Berbers (Hamosemites) on the highest higherarchial species level.

**Fig. 5:**
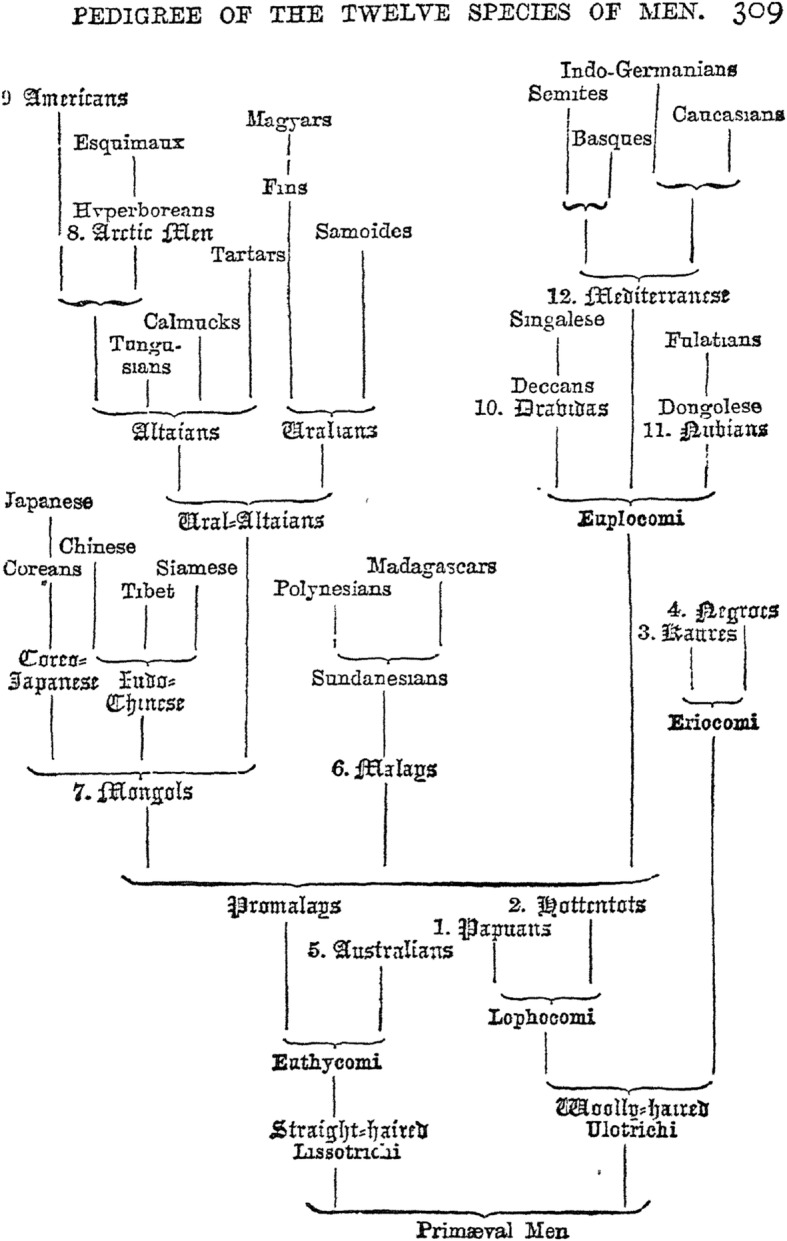
English version of the human pedigree of the 12 species (from Haeckel, The History of Creation, 1887, p. 309)

**Fig. 6:**
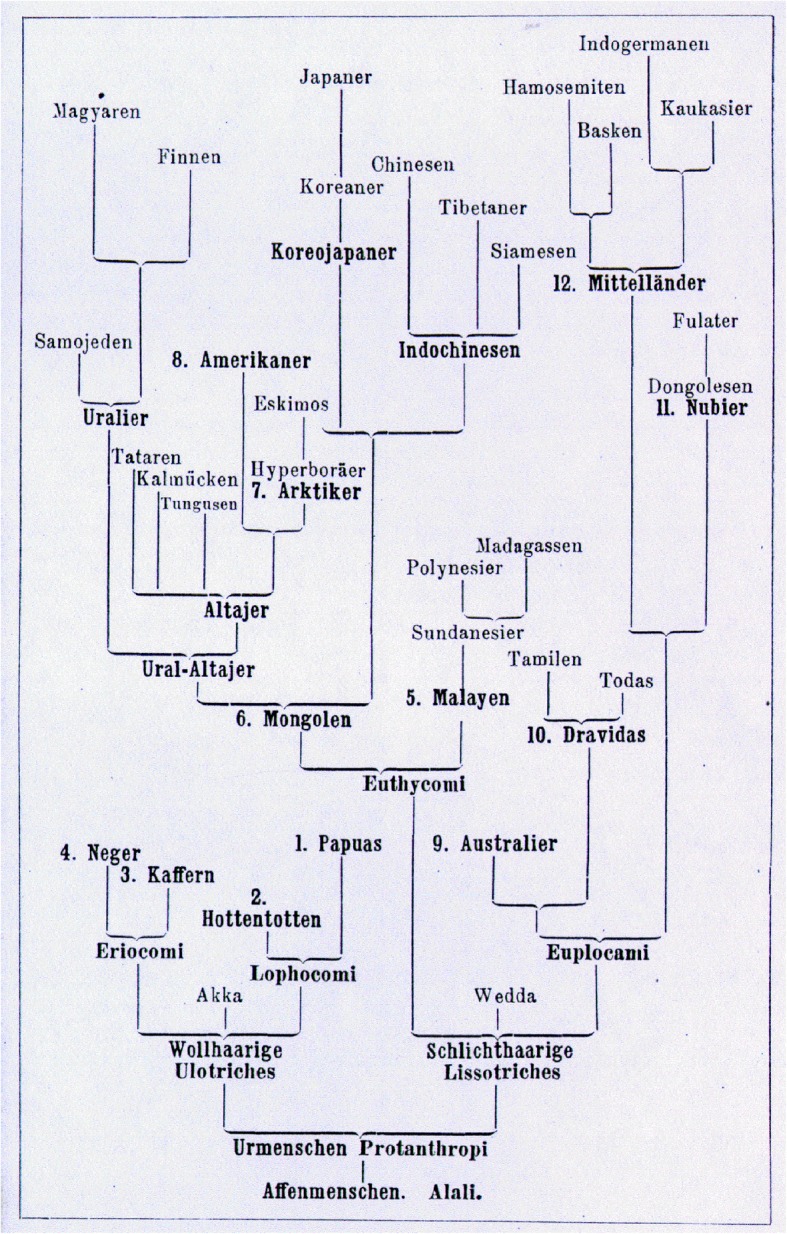
Genealogical-tree with 12 human species and minor races (Natürliche Schöpfungsgeschichte, 1889, p. 727)

Yet, from the contemporary viewpoint, Haeckel’s “speculations” (his own words) are definitely racist in the strict technical sense, since he admitted that the Earth, still in his own time, was populated by several human races so different from one another that they may even be considered to be different species positionable within a hierarchy. Again, Haeckel had only a few fossil data at his disposal, and reaffirmed continuously that his “trees” were provisional in nature. It is certainly true that (and in contrast to Darwin) Haeckel overemphasized progressive evolution, and that his diagrams were at the core of his attempts to explain his evolutionary paradigm [6] (Figs. 5 and 6). But the genuine intention of Haeckel’s racial diagrams was not to create a ready-made discriminatory scheme of hierarchical relations between human species, but rather to suggest hypotheses moving biological anthropology towards an exact Darwinian science based on comparative anatomy, phylogenetic and ontogenetic studies [20, 21, 40]. Where Darwin was cautious in making definitive claims, Haeckel rushed to establishing a new phylogeny of human species, and to prove the very fact of human evolution. The shortest way to prove it would be to demonstrate that even currently living humans are at various evolutionary stages. This led to a crucial discrepancy between Darwin and Haeckel: “As proponents of common descent, both had to reject polygenism in favor of monogenism, but they differed in how far back they would place the last common ancestor of all the races and whether they would count it as already human” [9]. Haeckel placed the last common ancestor further back in time than Darwin.

Systematic efforts to establish Darwinian anthropology were already made by Haeckel’s first scientific account of Darwinism, in the double-volumed *Generelle Morphologie* (Haeckel 1866). Here Haeckel clearly stated that anthropology becomes a science only in the light of evolutionary biology. He defined anthropology as a general biological science of humans, and a branch of zoology subdivided into human morphology and human physiology. Two years later in his *Natürliche Schöpfungsgeschichte* (German original published first in 1868; in English translation, e.g.: *The History of Creation*, Vol. I 1880; Vol. II 1887) Haeckel clearly stated that the origin of man can ultimately be traced back to *Monera,* and specifically that “Man has developed gradually, and step by step, out of the lower Vertebrata, and more immediately out of Ape-like Mammals” [15]. He also introduced the idea of *Pithecanthropus alalus* (speechless man) originating from Asian man-like apes (*Catarrhini*) as a hypothetical link between *Anthropoides* and the actual “speaking- or genuine man” (*Homines*) [15, 32]. Human origin from *Catarrhini* was secured by the argument that the anatomical differences between Man and the most human-like apes (Orangutan, Gorilla, Chimpanzee) are less than the differences between the latter and the lowest stages of *Catarrhini* such as Baboon. Altogether a human ancestral ladder („Ahnenreihe des Menschen“) consisted of 22 steps with *Pithecanthropus* in 21^st^ place and “genuine humans” on top. Aboriginal Australians and Papuans were categorized by Haeckel as the nearest living relatives of the ancestral stages.

Humanity consisted for Haeckel of 12 species, subdivided into 36 races. The human species were, among others, presented in their pedigree hierarchically from “lower” (more like apes) to “higher” (higher mental development), with Indo-Germanians on the highest level, and Hottentots, Papuans and Aboriginal Australians in the lowest part of the pedigree. Haeckel’s genuine motivation for arranging human races into “lower-higher” relations was his attempt to apply human genealogy as evidence for the animal origin of man. He looked for a resemblance “between the lowest woolly-haired men, and the highest man-like apes” [15] as evidence for human evolution. The latter led to the controversy between Haeckel and his Russian pupil Nikolai N. Miklucho-Maclay who, by way of empirical ethnographic studies in New Guinea, demonstrated the absence of significant differences between human races and the unity of all humans. Maclay spent several years among Papuans and came to the conclusion that both Haeckel’s morphological descriptions (for example, the character of their hair) and his estimations of their intellectual abilities were wrong.

**Fig. 7:**
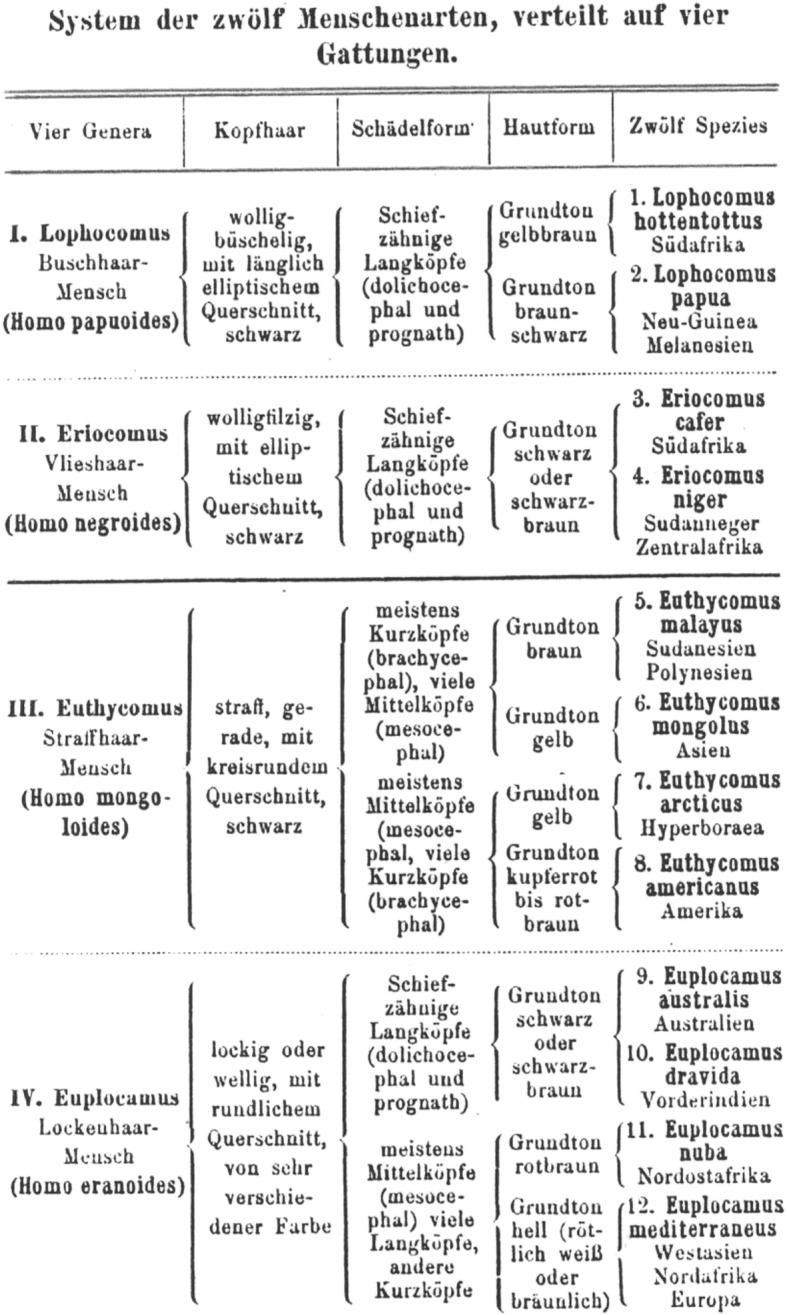
The System of 12 human species as a table. The Papuans belong here to the genus *Homo papuoides*, species *Lophocomus papua* (Natürliche Schöpfungsgeschichte, 1889, p. 742)

**Fig. 8:**
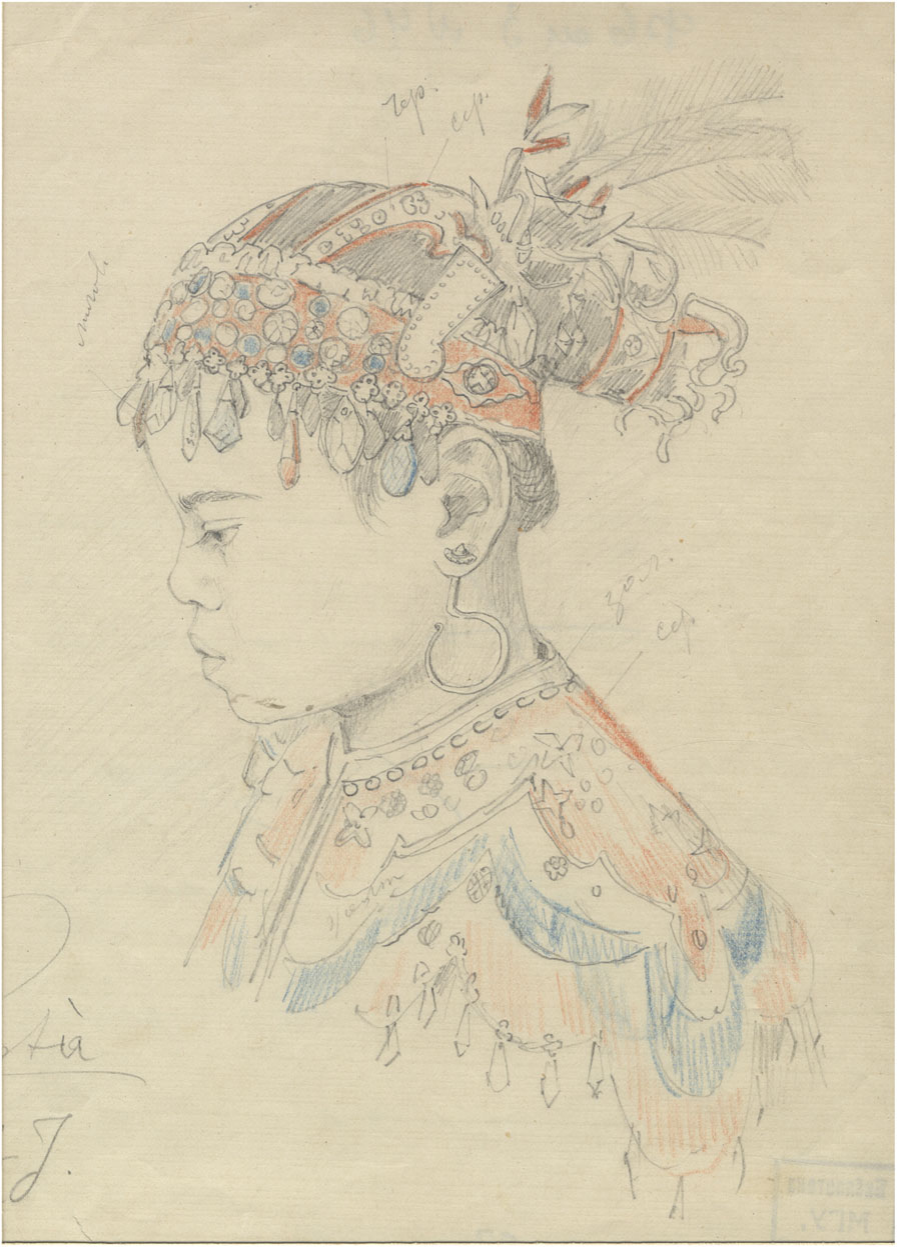
A young boy, 8,5 old. From Maclay’s expedition to the Malay Archipelago, Island Tidor. 1873–1875. RGO, file 6, List 3, № 46

**Fig. 9:**
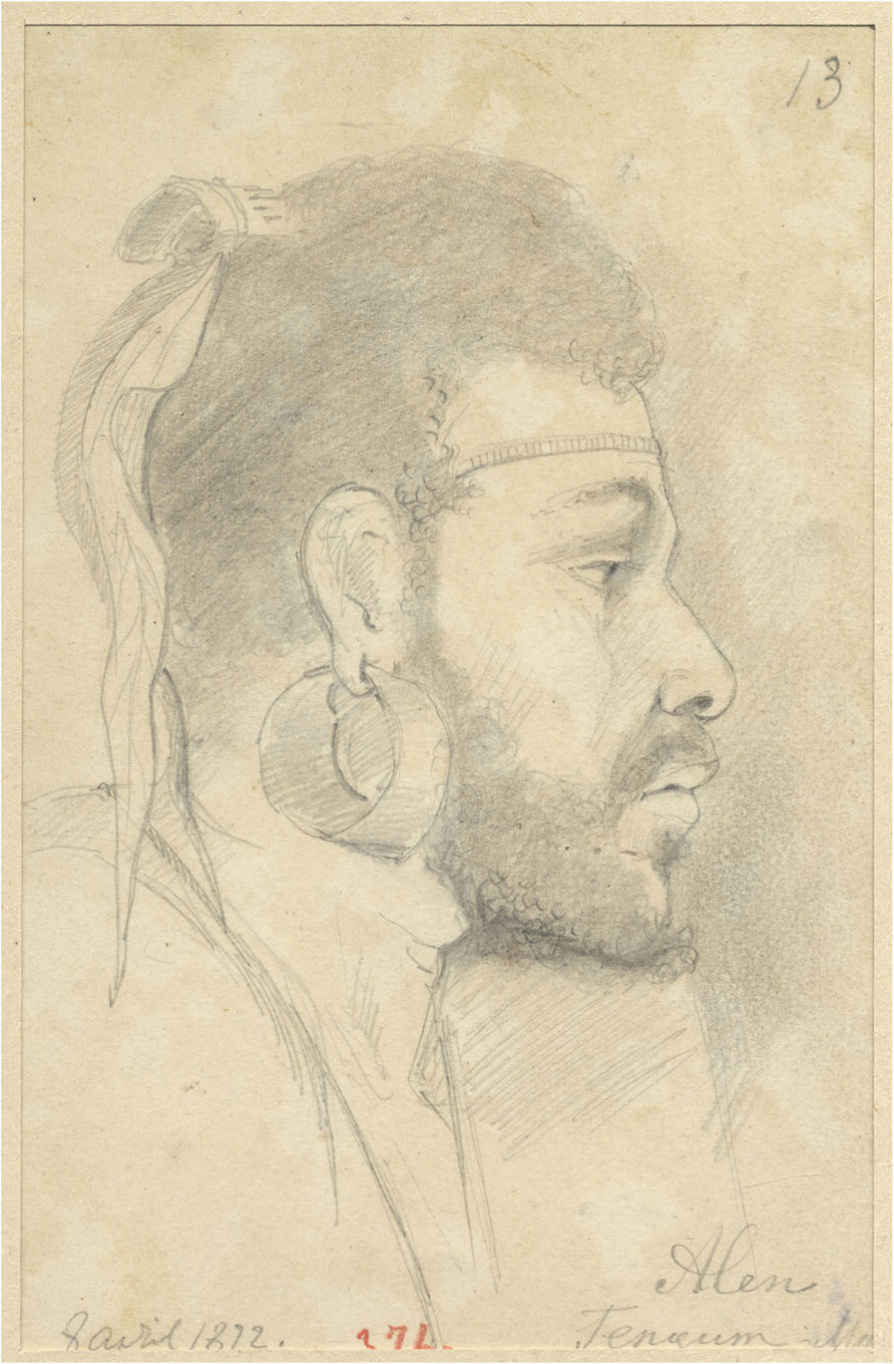
The portrait of the Papua male named Alen. The Maclay Coast. 1872. RGO, File 6, List 3, № 33

Despite believing in 12 human species co-existing on Earth (Fig. 7), Haeckel was not a genuine polygenist; he did not believe in the separate *creation* of human races, advocating instead a monophyletic origin of all human species on a hypothetical continent *Lemuria* in the Indian Ocean. In that sense he remained fully a Darwinian anthropologist. The major question for Haeckel, Darwin and other early anthropologists was not the monophyletic origin of man. The question was how far back they would place the last common ancestor of all human races or species, and therefore how human-like was this hypothetic ancestor? Haeckel tended to give human species more time to diverge (definitely more than Darwin), but they nevertheless diverged already around the human level. Considering Haeckel’s disregard of what was then a wide-spread racial prejudice, his views were intended to be progressive, given that he aimed to base his racial views on Darwinian science. The Papuans played a very special role in Haeckel’s story as they were in the lowest part of the pedigree and represented, so to say, an evolutionary dead-end. As will be shown below, the Papuans had a very significant place in the scientific objectives of Maclay as well [19, 49] (Figs. 8 and 9).

In the first edition of the *Natürliche Schöpfungsgeschichte* published in 1868 (i.e. written at the time when Maclay was studying in Jena) Haeckel already approached Papuans as a research subject. In the survey of the ten human species (in later publications he advocated 12 human species) and subspecies, Haeckel classified Papuans as *Hominis ulotriches* (wooly-haired men). The same category embraced *Homo primigenius* (Urmensch, prehistoric or primordial man, the same as *Pithecanthropus primigenius* or *Protanthropi*), *Homo hottentottus* (Hottentots), and *Homo afer* (black Africans) [13].

There are two hypotheses underlying this classification. First, that hair is a crucial character indicating someone’s place on the genealogical-tree. Haeckel was not the first and not the only to propose that hair is a distinctive feature to be used in human classifications. The idea was with all probability initially coined by Jean Bory de Saint Vincent (1778–1846) in 1825 [29] and was employed by both Haeckel and F. Müller. Haeckel developed his “hair-based” classification into a very detailed theory, where Papuans were labeled “bushy-haired” (Büschelhaarige) whereas, for example, Africans were categorized as “fleecy-haired” (Vlieshaarige), although all of them belonged to the same higher taxonomic category of “wooly-haired” humans [13, 15]. His descriptions of the “bushy-hair” of Papuans shows how little he knew about real Papuans: “Their woolly hair grows in tufts, is spirally twisted in screws, and often more than a foot in length, so that it forms a strong woolly wig, which stands far out from the head” [15]. Haeckel’s attention to the hair structure partly explains the importance of this character for Maclay, and his excitement when he discovered that Papuans’ hair is not “bushy”. Maclay thus made a step towards current view that abandonded hair structure and skin color as racial discriminatory features [7].

Second, Papuans, for Haeckel, belonged to the most primitive group of human species. The most primitive means here that they were supposed to be phylogenetically closer to the pre-human hominids. Geographically, Haeckel placed Papuans in the region of New Guinea, New Britain (an island in the Bismarck Archipelago), New Caledonia, New Hebrides, and the Solomon Islands. The Papuans (a species *Homo papua* or *Lophocomus papua*) were for him “most closely related to the original primary form of wooly-haired men” of all still living human species [15]. Once again, even in the 1880s, after Maclay’s reports were already published in the most respectable scholarly journal *Nature*, Haeckel continued to insist that Papuans belong to a special human species: “The peculiar form of their hair and speech, so essentially distinguishes the Papuans from their straight-haired neighbors, from the Malays as well as from the Australians, that they must be regarded as an entirely distinct species” [15]. As to the state of their development Haeckel made controversial statements. In 1868 he claimed that some of them are “open to culture” to a relatively high grade, whereas some of them remain on the “lowest level” of development among other humans [13]. At the same time, Haeckel clearly stated that his classifications presuppose the distinction of humans into higher and lower categories and that this classification is of biological nature. He insisted that the Aboriginal Australians and “some tribes of the Polynesians” (Papuans) are at the lowest stage of human mental development as well as “the Bushmen, Hottentots, and some of the Negro tribes” [13, 15].

Haeckel argued that the language of Papuans, their arithmetical abilities and culture are at the lowest level as they are incapable of the simplest abstractions, although these claims were completely disproved by Maclay in the 1870s and 1880s. The lifestyle of Papuans, Haeckel asserted, is closer to that of apes than to that of civilized men. Most importantly, Haeckel concluded that this human species cannot be civilized in principle, because the Papuans’ brain remained evolutionarily underdeveloped: “All attempts to introduce civilization among these, and many of the other tribes of the lowest human species, have hitherto been of no avail; it is impossible to implant human culture where the requisite soil, namely, the perfecting of the brain, is wanting” [15]. Human species, according to Haeckel, evolved under the adaptive pressure and, among others, due to Darwinian natural selection: “The theory of selection teaches that in human life, as in animal and plant life everywhere, and at all times, only a small and chosen minority can exist and flourish, while the enormous majority starve and perish miserably and more or less prematurely” [14]. However, in his view, human evolution was tending largely, but not exclusively, in a ‘progressive’ direction. Thus, for example, for Haeckel the “low stage of mental development” of Aboriginal Australians must have arisen from *degeneration*, “that is by adaptation to the very unfavorable conditions of existence in Australia” [15]. Haeckel’s view that there are higher and lower developed human species co-existing on Earth was a result of combining the struggle for existence within natural selection and his speculative concepts concerning phylogenies. Guided by the idea of the struggle for existence Haeckel arrived at the conclusion that Papuans, as well as other “underdeveloped” human species, would sooner or later disappear from the Earth’s surface, being ousted by the more advanced human beings [13].

Haeckel speculated about the race issues and the role of Papuans in the racial hierarchy until his very last days. In a small book published shortly before his death *Ewigkeit: Weltkriegsgedanken über Leben und Tod, Religion und Entwicklungslehre* (1915) [*Eternity: World War Time Thoughts about Life and Death, Religion and Evolutionary Theory*] written at the beginning of the First World War, Haeckel in a polemic style repeated his thesis that “black-brown” Australians and Papuans represent lower human races [*niedere Menschenrassen*] [17]. He stated again, as in the first edition of the *Natürliche Schöpfungsgeschichte* in 1868, that the difference between “highly developed European nations and the lowest savages” is more significant than that between “the savages” and anthropoid apes. Thus, despite the growing evidence of the contrary, Haeckel never gave up the idea of subdividing humans in lower and higher races in terms of their place on the genealogical-tree. At the same time, Haeckel was no “desk theoretician”. During his ca. fifty years in Jena, he travelled a lot, 1859/60 to Italy, 1866 to the Canary Islands, 1866 to Norway, 1870 to the Orient, 1875 to Corsica, 1876 to Scotland, 1878 to Brittany, 1887 again to the Orient, 1890 to Algiers, and 1897 to Russia. In addition to these “short trips”, Haeckel visited tropical regions (in 1881 Ceylon; nine years later, Java and Sumatra), where he not only explored nature, but was also an active artist. But even after his long-term expeditions to tropical islands, where he directly contacted native populations, Haeckel did not change his mind and continued to think in terms of lower and higher races [16].

Meanwhile, as Haeckel continued to speculate about higher and lower humans, his Russian pupil Miklucho-Maclay was occupied with the thorough field studies that challenged the very foundations of Haeckel’s human phylogenetic trees. The major point of controversy were the Papuans.

**Fig. 10:**
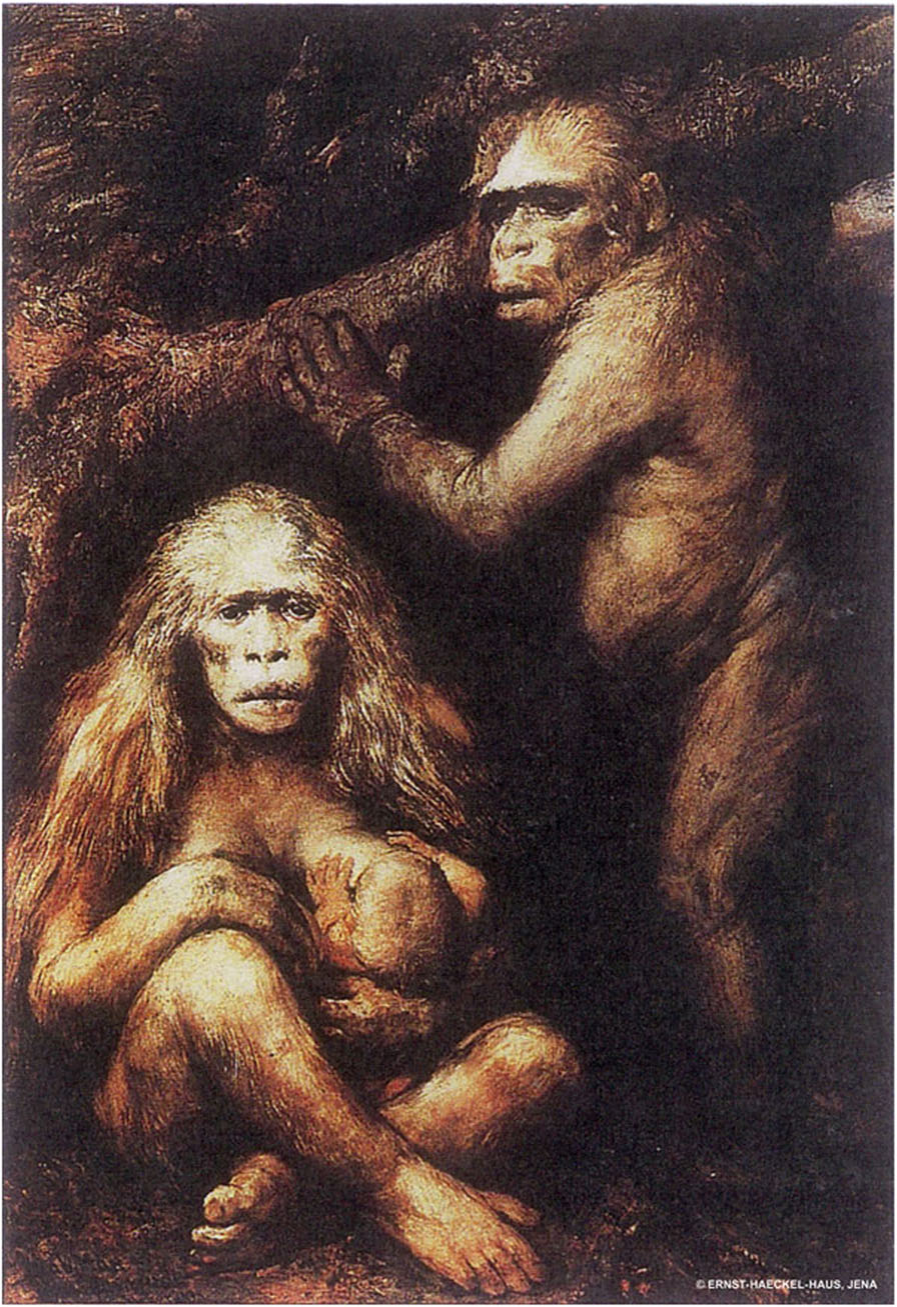
Gabriel von Max: Pithecanthropus alalus (1894), Oil painting. Ernst-Haeckel-Haus in Jena (EHH-Archive, Jena)

To sum up, Haeckel developed a hypothesis claiming that the current Earth is populated by several human species, some of which are phylogenetically closer to the common human ancestor *Pithecanthropus alalus,* whereas other species are at a much more advanced stage of evolutionary development (Fig. 10). Cultural evolution and biological advancements were going hand in hand, enhancing each other during human evolution. This picture, Haeckel hoped, would contribute to prove the very idea of human origins from an ape-like ancestors. Papuans were for Haeckel a kind of an intermediate link between *Protanthropi* man and modern Europeans.

As one of the lowest human species, Papuans played a special role in the story as their morphological characteristics and – in Haeckel’s mind - underdeveloped brain should have been living proof of evolutionary progress of more advanced human species. This was exactly the point where Maclay intervened into the Haeckelian theory by derailing his idea that Papuans demonstrate specific morphological and linguistic features that would “prove” their lower evolutionary stage in comparison to European nations. By attacking Haeckel’s grasp of Papuans, Maclay jeopardized the whole human phylogenetic tree proposed by Haeckel.

As we will see below, Maclay adapted from Haeckel and other contemporary Darwinians the central methodological idea that races are natural phenomena to be approached empirically. It was evident for him that the science of races can be built into a more general picture of growing (Darwinian) anthropology and allied sciences. Yet Maclay was not ready to accept the idea of humanity consisting of various hierarchically structured species. His intention was not to design speculative anthropological theories as Kant and Blumenbach had done, but to apply the very empirical method, which he learned in the Jena period primarily under the guidance of Haeckel [19]. At the core of this method was not only the general idea of evolution, the method of detecting geographic variations by appealing to anatomical and physiological characteristics, but also the Haeckelian way of exact observations of organisms in their natural environments. The connections and interactions between organisms, their place in the environment, were so important for Haeckel, that he coined the term “ecology” to describe it [67, 70].

### Nikolai Miklucho-Maclay: a biographical note

**Fig. 11:**
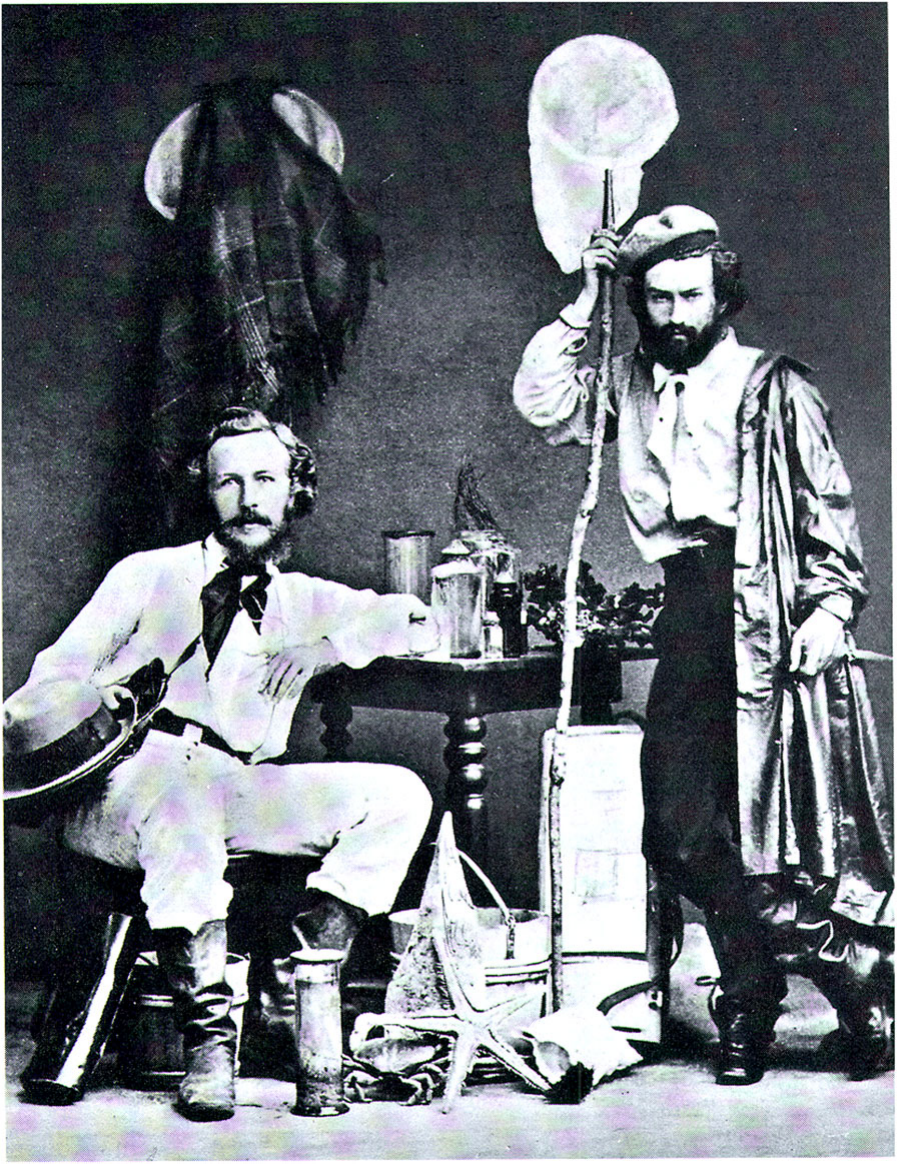
Haeckel and Maclay before their trip to Canary Island (Lanzarote) 1866 (RGO St. Petersburg)

As Miklucho-Maclay is little known outside of Russia, and there are just a few biographical sources available in English, we summarize here relevant biographical details (Fig. 11).

Nikolai Nikolajevich Miklucho was born on 17 July 1846 (Gregorian Calendar) in the Gouvernement Nowgorod of the Russian Empire. Nevertheless, he always emphasized his origin from Ukrainian Cossacks, which may be explained by his early interest in ethnic issues. His father was a railway engineer, who received a nobility title for his services; this allowed Nikolai to call himself “von Miklucho” when in Germany. After his father’s death in 1857, the family grew impoverished; from that period on his financial problems were his permanent companions [63]. Already as a high school student, Maclay was fascinated by Russian liberal thinkers such as Alexander Herzen (1812–1870), Nikolai Dobrolyubov (1836–1861), Nikolai Tschernyschewsky (1828–1889) and others who stood in opposition to the Tsarist regime, and who spread progressive and humanistic ideas. Russian liberalism at that time was tightly coupled with the admiration of natural science. Ivan Turgenev’s famous novel “Fathers and Sons” (1862) captured this link between natural science and political views by addressing the problem of generational change in Russia. Turgenev and Maclay met several times in 1869 [63]. Alexander Herzen published in 1845/1846 in a popular literary journal *Otechestvennye Zapiski* (Domestic Notes) a philosophical essay “Letters on the Study of Nature”, where he, among others, emphasized the importance of empirical knowledge, and even speculated about the methods of morphology [30]. A radical social thinker and literary critic Dmitry Pisarev (1840–1868), who was very influential among liberal students, published in 1864 in the journal *Russkoje Slovo* [Russian Word] the voluminous paper “Progress in the Realm of Animals and Plants”, in which he made Darwin’s *Origin* accessible to a broad audience, explaining it chapter by chapter [36].

In 1863 Maclay dropped out of his high school (Gymnasium). There is a hypothesis that his liberal anti-Tsarist views played a significant role in this. The same year he enrolled in the Physical-Mathematical Department of the St. Petersburg University as a so-called “guest student”, which meant that he was allowed to attend lectures without being formally admitted as a student. However, already in 1864, he was expelled from the university for violating the university rules by actively participating in students’ political actions. Because of a low probability to be allowed by authorities to enroll in another Russian university, Maclay decided to move to Germany, where no university entrance certificate was needed and no political repressions of Russian students existed. It is his German period, the student Miklucho became Miklucho-*Maclay*, although no ultimate explanation of this decision exists.

**Fig. 12:**
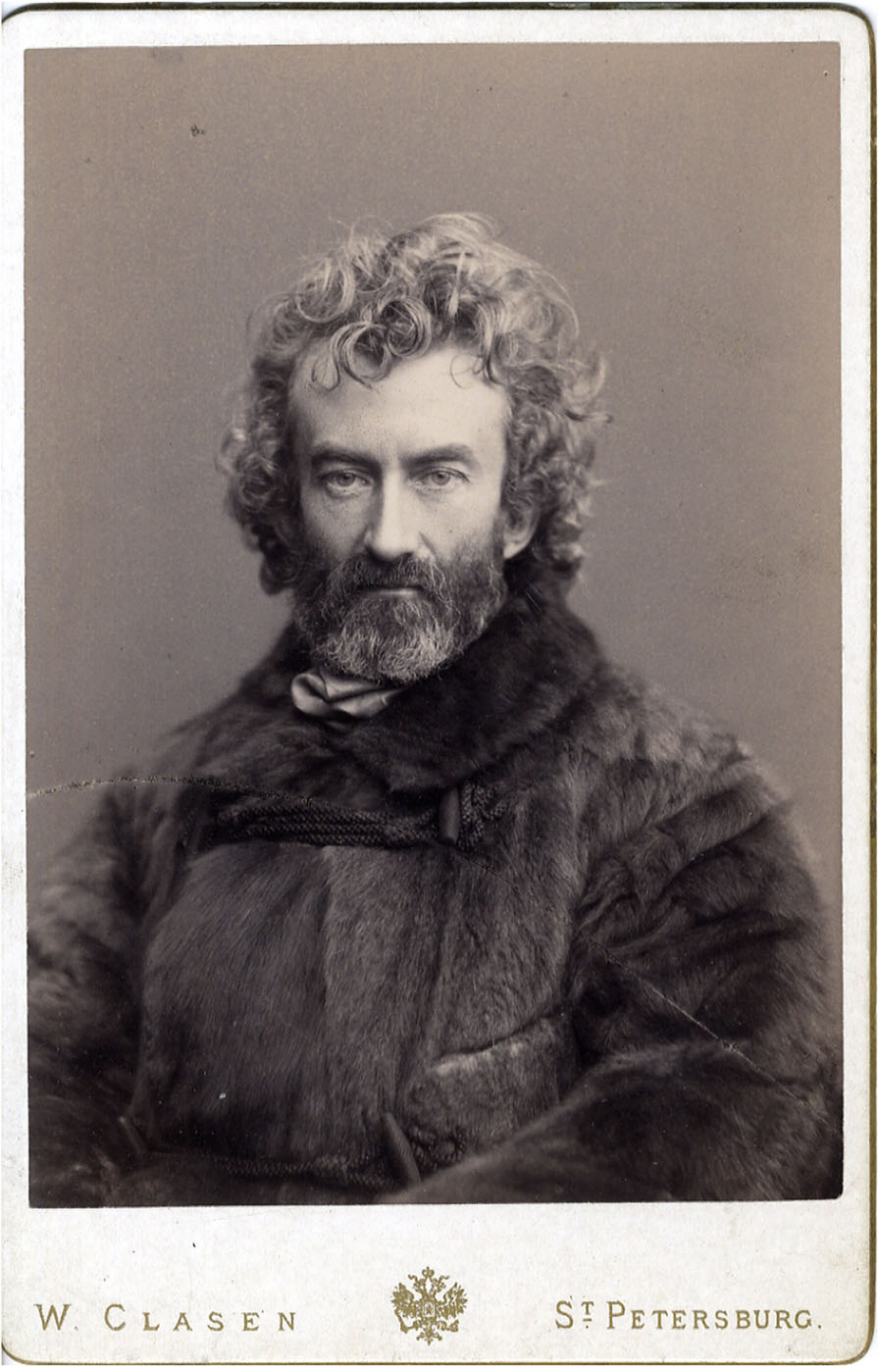
Мaclay in a fur coat. Winter 1886 in St. Petersburg. RGO, File 6, List 3, № 20

After spending a while at the Universities of Heidelberg and Leipzig, Maclay in October 1865 moved to Jena and a month later enrolled in the Medical Department of Jena University [6]. Between 1866 and 1868 Maclay studied zoology and anatomy with Haeckel and Haeckel’s senior colleague Carl Gegenbaur (1826–1903) and became Haeckel’s assistant [21, 39, 40, 65]. During this time Maclay very attentively listened to Haeckel’s and Gegenbaur’s lectures. His detailed and well-illustrated lecture notes are kept in the archive of the Russian Geographical Society in St. Petersburg [26, 27]. This was exactly the time when Haeckel gradually became a center of gravity of continental Darwinism due to public lectures and writings, but especially due to the publication in 1866 of his double-volume foundational opus, the *General Morphology*, wherein Haeckel’s version of Darwinism and his research method were made fully explicit and presented in detail [12]. From November 1866 to April 1867 Maclay accompanied Haeckel on his research expedition to the Canary Islands via Switzerland, France, Spain, Portugal, and Tenerife. The ultimate objective of the expedition was to study sponges and brains of cartilaginous fishes on the island Lanzarote [44]. Remarkably this expedition took place after Haeckel had visited Charles Darwin at Down House on October 21st, 1866, so that Maclay was with all probability informed about this historical event. For him, the Canary studies resulted in a publication in German on the comparative neurology of vertebrates, where Maclay applied the descriptive techniques and research method acquired in Jena [43] (Fig. 12).

One of the best-known photographs of Haeckel is his joint portrait with Maclay; it was taken in Jena at the time of their Canary trip. This joint expedition is of special importance because it brought Haeckel and Miklucho in a very close contact, making the latter into Haeckel’s close associate for a while. In 1869 Maclay returned to Russia, and in 1870 he began preparing his first expedition to Polynesia [44]. Beginning in 1871, Maclay broke all communication with Haeckel. Even his monograph of 1870 was devoted to Carl Gegenbaur, and not Haeckel (as one would expect). One of the possible reasons for their alienation from each other was their growing disagreement over the nature of the human race [62]. The very last letter to Haeckel signed with the name Miklucho-Maclay was written by his Australian widow Margaret after his death in 1893. In this letter, written in English, she assured Haeckel that Nikolai Maclay held “deepest feelings of esteem and regard” toward Haeckel and called him Haeckel’s “old friend in science”. The major objective of Margaret’s writing was to win Haeckel’s support for the publication of Maclay’s scientific legacy. Despite its pragmatic character, this letter is a testimony of the deep influence of Haeckel on Maclay.

Back in St. Petersburg Maclay began with the preparation of his expedition to New Guinea and on September 27th, 1869, he submitted his initial plan to the *Imperial Russian Geographical Society* (RGO). In the same year, he received a research grant from the RGO and permission from the Tsar Alexander the II to join the corvette “Vitjaz” (Knight) to reach New Guinea [44]. Yet before his departure, Maclay visited Jena one more time (1869–1870) to finalize the publication of his first monograph. He also spent some time in Berlin, Leiden, Rotterdam, Brussels, and London in order to strengthen scientific cooperation with leading scholars. In October 1870 he presented his detailed research program to the RGO and at the end of the same month left for New Guinea. Altogether Miklucho spent about 17 years in the tropic regions, including six expeditions to New Guinea (1871–72, 1874, 1876–77, 1880, 1881, 1883) [52].

**Fig. 13:**
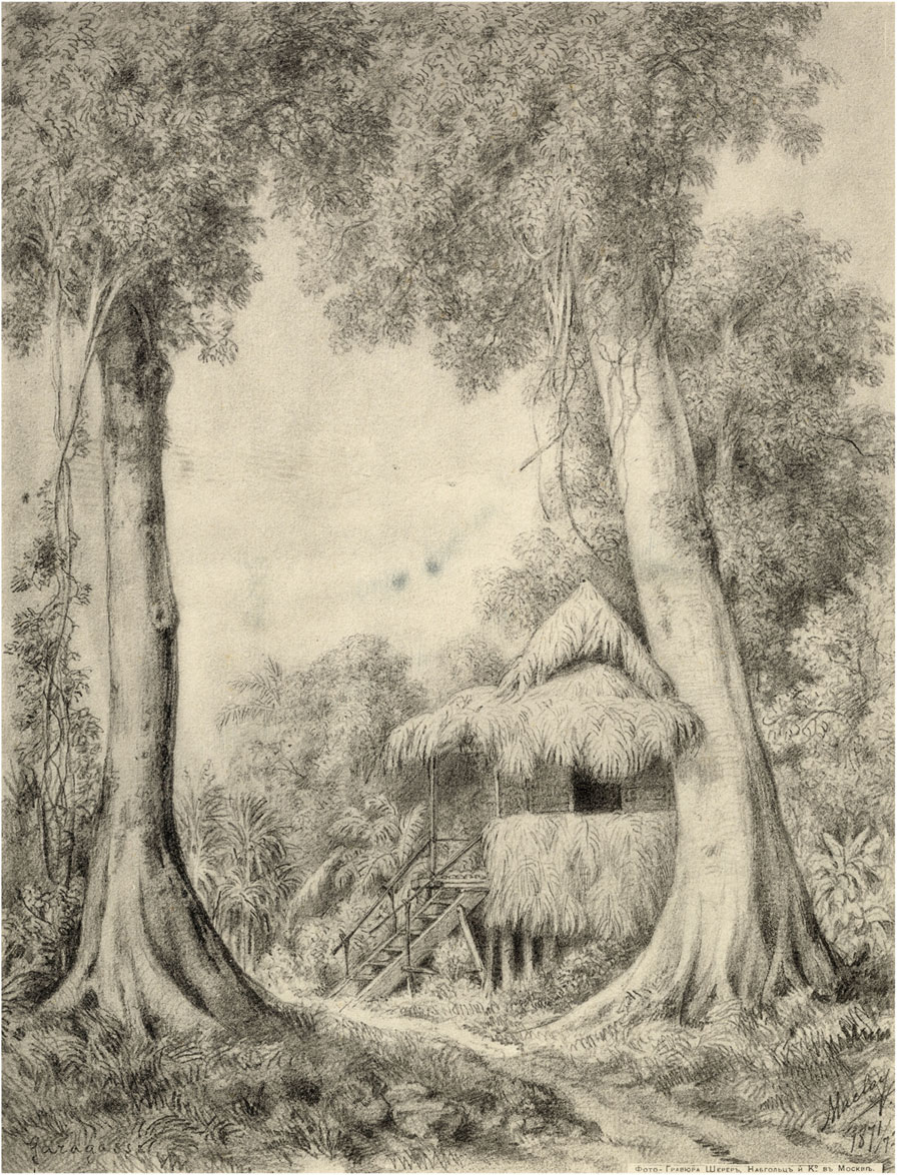
Maclay‘s first hut in Garagasi, New Guinea 1871–1872. RGO, File 6, List 3, № 33

**Fig. 14:**
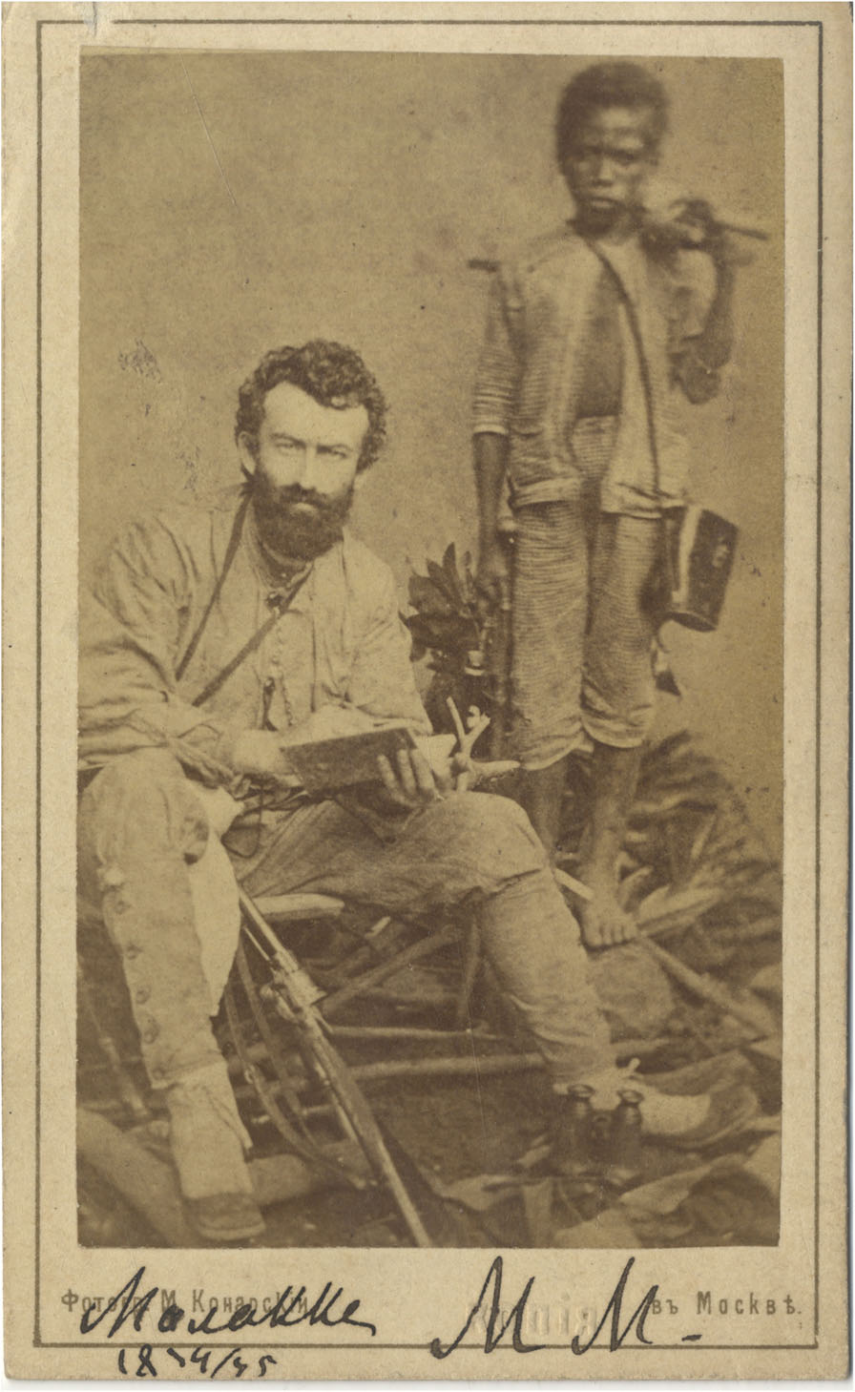
Maclay with his assistant Akhmat. 1874–1875. RGO, File 6, List 3, № 20

The major funding came from the Imperial Russian Geographical Society, but some financial support was offered also by the Anthropological Society of Berlin (Berliner Anthropologische Gesellschaft) founded by the famous German anatomist and anthropologist Rudolf Virchow (1821–1902) with whom Maclay was in contact, and who helped Maclay with publications. Maclay had an opportunity to conduct long term stationary research in New Guinea and other regions of Polynesia and Melanesia (Fig. 13). Only in 1871–1872, Maclay stayed on the north-east coast of New Guinea for an uninterrupted 15 months [11]. One of the major objectives of this expedition was an anthropological and ethnological study of Papuans [44] (Fig. 14). Maclay traveled a lot throughout the world, visiting many countries that, for European travelers, were ‘exotic’: Singapore, Jakarta, “Dutch India” or Siam. Australia became his second homeland as in February 1884 he married Margaret Emma Robertson-Clark Robertson (1855–1936), the daughter of the President of New South Wales, John Robertson (1816–1891). Maclay was politically very active throughout his whole life, trying to apply his knowledge of Papuans and other native folks to protect them from slavery and exploitation by industrial nations. He enjoyed a very high level of confidence from indigenous populations; the Papuans called him Kaaram-tamo, “The Moonman” partly on account of the supernatural capacities they ascribed to him [46]. To this day the natives of New Guinea’s Maclay Coast cherish the memories of him, and the name Nikolai is frequently given there.

Maclay died on 14 April 1888 in St. Petersburg without being able to systematize his scientific legacy, which was partly lost and destroyed. His works were organized and prepared for publications by his biographers and researchers, in recent times by such authors as Boris Putilov, Nikolai Butinov, and Dmitry Tumarkin.

### Miklucho-Maclay’s race studies

The major problem with describing Maclay’s anthropological and ethnological views is his predominantly descriptive way of presenting scientific results. He avoided sweeping generalizations and, in that sense, was the exact opposite of Haeckel who tended to engage in broad theoretical speculation. Yet, Maclay’s anthropological and ethnographic observations were designed in such a way that they approached burning issues of his time. They are of a high value even now, because he arrived in New Guinea when the culture of Papuans was of a “purely” Neolithic character, without any external influences. His program of studies was very interdisciplinary but included anthropological questions as its very important elements. To achieve his goals Maclay developed a method of “stationary fieldwork”. Maclay learned Papuan language and beliefs which allowed him to live among the indigenous popluations, conducting observations and anthropological measurements from both etic and emic perspectives, thus acquiring information inaccessible for customary researches. For example, he even succeeded in measuring an erected penis of a Papuan. As *Nature* reported in 1882: “When in search of a place at which to study the customs and life of the primitive people at the lowest stage of culture, M. Maclay chose the north-western coast of New Guinea, close by Astrolabe Bay, which was never visited before by Europeans. [ …] He built his hut between two Papuans villages, on a promontory that was occupied by nobody” [46].

At the same time, being intimately involved with the natives, Maclay was far from being just a sentimental travelling diarist. In 1876 he emphasized that during his first expedition to New Guinea in 1871–72 he “studied Papuans rather from anthropological (anatomical) viewpoint” [44]. His notes reveal an application of a scientific method, and that method was to a great extent acquired by him while studying zoology and medicine in Jena: “Occupation with comparative anatomy (especially with sponges) convinced him of the importance of geographical variation of animals [ …]. In that sense, Miklucho followed the most advanced tendencies of contemporary science. Like his mentor E. Haeckel, he considered racial variation from the same viewpoint as a variation of other biological species. It is not surprising that in the commented text we find notions like adaptation and inheritance. Racial modifications are seen by him [Maclay] as resulting from instant influences of social and natural environments” (A.N. Anfertjev in: 44).

To appreciate Maclay’s debt to Haeckel one must remember that for Haeckel certain anatomical features indicated a phylogenetic history of human species [40]. Along with the shape of the hair, Haeckel, as well as Rudolf Virchow and many other physical anthropologists at that time, paid a lot of attention to cranial geometry [19]. For example, Haeckel connected the head form of African apes and African human races and contrasted them to Asian apes and Asian human populations: “For it is a very remarkable fact, that the African man-like apes (gorilla and chimpanzee) are characterized by a distinctly long-headed, or dolichocephalous, form of skull, like the human species peculiar to Africa (Hottentots, Caffres, Negroes, Nubians). On the other hand, the Asiatic man-like apes (especially the small and large orang), by their distinct, short-headed, or brachycephalous, a form of skull agree -with human species especially characteristic of Asia (Mongols and Malays). Hence, one might be tempted to derive the latter (the Asiatic man-like and primeval men) from a common form of brachycephalous ape and the former (the African man-like apes and primeval men) from a common dolichocephalous form of ape” [15]. The hypothetical primeval continent Lemuria mediated these two extremes: “In any case, tropical Africa and southern Asia (and between them Lemuria, which formerly connected them) are those portions of the earth which deserve the first consideration in the discussion as to the primaeval home of the human race” [15].

Maclay disproved the hypothesis of a special hair-structure of Papuans (“bushy-hairiness”) as a reliable racial characteristic. John Galton reported in 1874 in *Nature* on Maclay’s findings: “After a series of very careful observations, made as well upon shaven as upon well-covered scalps, Dr. Maclay concludes that the hair is not naturally disposed, as has been represented, in tufts or clumps, but grows just as it would upon the head of a European” [8]. In a manuscript summarizing his expeditions of 1876 Maclay repeated this thesis and made explicit references to Theodor Waitz’ the *Anthropologie der Naturvölker* [*The anthropology of peoples that live close to nature*], Fritz Müller’s *Allgemeine Ethnographie* and Haeckel’s *Natürliche Schöpfungsgeschichte* as the sources of the “bushy-hairiness” hypothesis. “No geographic variety” of Papuans, Maclay argued, demonstrated that kind of hair [45].

Already in 1871–72, Maclay discovered that Haeckel’s hypothesis concerning Papuans’ skin was false as well: “a special design of Papuans’ skin can in no way be specified as a character differing them from other humans” [41]. Papuans skin color and design were not “of special character” as predicted by Haeckel and other anthropologists and could not be used for distinguishing Melanesians from other human “species”: “Black skin is not a feature characterizing the whole tribe [Stamm] of Papuans”, Maclay claimed.

Haeckel’s and Virchow’s idea that a certain race can be ascribed a tendency to dolichocephaly or brachycephaly was ultimately disproved by Maclay as well. After conducting hundreds of measurements and craniological studies on male and female Papuans, he concluded that “the long-headedness (dolichocephaly) does not hold as a racial characteristic of Papuans,” because among hundreds of heads with dolichocephaly he found dozens of brachiocephalic individuals [45]. In sum, Maclay’s “mere observations” quickly disproved a series of anthropological concepts formulated by leading scientists, Haeckel among them.

Maclay was fully aware of the significance of his descriptions already in the early 1870s. Commenting on his diaries of 1871–1872 Tumarkin remarked: “This diary clearly demonstrated Miklucho-Maclay’s worldview. The claim that Papuans were ‘bushy-haired’ was used by some anthropologists and biologists, including Ernst Haeckel, to substantiate the doctrine of racial inequality, placing Papuans as an intermediate link between anthropomorphic apes and Europeans. Being fully aware of the public danger of such theories Miklucho-Maclay has got a ‘good mood’ as he discovered that the arguments based on the bushy-hairiness of Papuans are erroneous” (Comment of D. Tumarkin in: 44).

Maclay’s observations were a result of thoroughly planned scientific expeditions and the research questions he posed were formulated in direct communication with leading scientists of his time. In his “Program of the Proposed Studies on the Islands and Coastline of the Pacific Ocean” announced to the Russian Geographical Society on the 7th of October 1870, Maclay numbered several scientists who directly influenced his research program. It included physical geography, meteorology, ethnography, anthropology, and political economy. The general objective of the expedition, which was initially planned to last seven to eight years, was to describe how organisms vary depending on varying environmental conditions [45]. This was quite a typical question for the Darwinian evolutionists of those days. Concerning specifically ethnography and anthropology, Maclay mentioned, among others, the detailed recommendations provided to him by a German ethnographer Adolf Bastian (1926–1905), a letter from a German geographer and anthropologist Georg Gerland (1833–1919), and a letter from a German-Russian universal naturalist Karl Ernst von Baer (1792–1876). Maclay was personally well acquainted with Baer and cited Baer’s publication *On Papuans and Alfuros* [1] directly related to the subject matter. Darwin was certainly one of Maclay’s inspirations as well, but there were no direct contacts between them, though he used Darwin’s published instructions for an Austrian expedition to South America and Eastern Asia. Rudolf Virchow also played a special role for Maclay, as he helped the young researcher to publish his studies. In addition, important for Maclay were verbal communications with the famous “Darwin’s bulldog” Thomas Henry Huxley (1825–1895), the comparative anatomist Carl Gegenbaur, anatomist and ethnographer Robert Hartmann (1832–1893), and “Darwin’s shadow man” Alfred Russel Wallace (1823–1913), who mentioned *Papuans and Alfuros* in the 2nd volume of his famous *The Malay Archipelago* [66]. The pre-Darwinian evolutionist James C. Prichard (1786–1848) is regarded as one of Maclay’s important influences as well [3]. Prichard championed the idea of the unity of human species (monogenism) understanding ethnology as a “natural history of man” [50, 51] and was one of the earliest scientific advocates of aborigines’ rights.

To return to Haeckel, he proposed Maclay pay attention to the following issues:

1. Histological structure of the head epidermis and other body parts;

2. The length of the big toe and of the male organ; female’s glute shape and female genitals and breast; teeth of various races.

3. Swimming of various inhabitants of the Pacific islands (how do they swim) [45].

As Maclay’s diaries document, he followed Haeckel’s suggestions quite exactly and investigated even the most intimate details of Papuans’ anatomy and physiology.

**Fig. 15:**
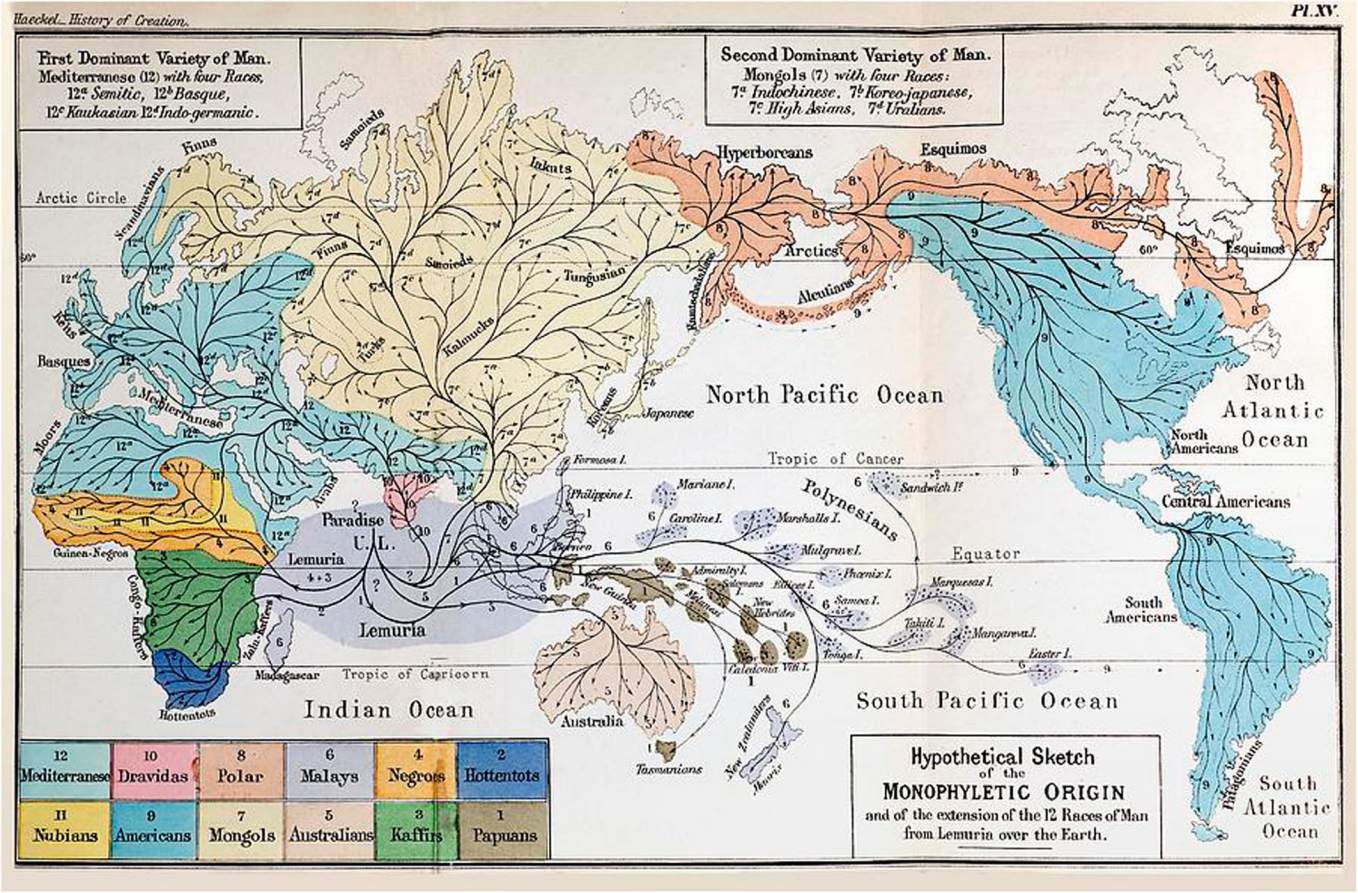
The Map of Lemuria. Hypothetical sketch of the monphyletic origin and the diffusion of the twelve human species over the Earth (The History of Creation, 1887, Plate XV)

The idea that Maclay’s choice of New Guinea as his major research place “was made under the influence of his mentor Ernst Haeckel”, was first expressed by Butinov [4] and then reintroduced by Belkov [3]. The hypothesis proved that Haeckel’s concept of a hypothetical continent *Lemuria*, which once existed in the Indian Ocean, and which served as the cradle of humanity, was an important factor in Maclay’s decision to study the Papuans. Although Wallace did not exclude the existence of Lemuria [56], it was Haeckel who passionately pushed this idea forward, making it into the cornerstone of his evolutionary anthropology [33, 34] (Fig. 15).

Haeckel, indeed, proceeded from the assumption that immediate predecessors of modern humans evolved from anthropoid apes on the “primordial continent” Lemuria, extending between the Sunda-Islands and the African East-coast [13]. Haeckel called the human species evolved on Lemuria *Homo primigenius* [69]. The Papuans were, according to Haeckel, the living remnants closest to the original form of the wooly-haired man [13]. Geographically, New Guinea is close to the Eastern Shore of the hypothetical continent and it was tempting for Haeckel to formulate a hypothesis that the human species geographically closest to Lemuria was the most primitive one [69].

It is important to appreciate the pivotal role of Maclay’s choice in terms of Haeckel’s speculative system. Disproving the hypothesis that Papuans were the most primitive human species with distinctive morphological traits would overturn the whole Haeckelian human phylogeny. This proved strong enough motivation for a young ambitious naturalist to go to New Guinea. Maclay was explicit about his intentions to investigate New Guinea as the hypothetical homeland of Haeckel’s “primordial man” [61]. Belkov has no doubts that Maclay’s plan matured in Jena under Haeckel’s direct influence: “If one considers that information taken from Haeckel’s lectures overlapped in Miklucho-Maclay’s mind with the Gerland’s ethnic map of the region, in which all areas listed by Haeckel are brown-painted as the Papuans settlements, there would be no doubts how and where he came to the idea of expedition to New Guinea to study the Papuans” [3]. In a short, uncompleted note presumably written in 1871–72 [4] with the title “Why I Chose New Guinea as a Field of my Studies” Maclay, indeed, mentioned “problematic continent, so called *Lemurii*” in the context of discussing the importance of New Guinea for exploring the nature of Polynesia [45]. Maclay looked for Haeckel’s primary human species not only in New Guinea, but also on the Philippines (1873), Malaki (1874–1875), and various Melanesian islands [4]. He found nothing close to Haeckel’s descriptions.

Maclay conducted thousands of exact and very detailed observations and measurements of anatomical, physiological, linguistic and cultural kind. His conclusions were based on this titanic, single-person fieldwork, and not on theoretical speculations as in Haeckel’s case. For example, in February 1877 Rudolf Virchow received a letter from Maclay, which was accompanied by a manuscript describing the results of his nine-months long studies in West-Micronesia and North-Melanesia in 1876. In the letter, Maclay asked Virchow for support for his anatomical race-studies. Despite the transdisciplinary nature of Maclay’s methodology, anatomy belonged to the standard instruments of his research routine. He always stressed: “the imperative necessity of human races anatomy as a foundation of anthropology” [42]. Under “anatomy” Maclay understood techniques such as head- and skull measurements, descriptions of height, skin-color, rudiments of *Palpebra tertia*, the secondary sexual characteristics, big toe, hair-structure and in-depth investigations such as the brain anatomy.

And he conducted such measurements in statistically significant numbers. During his 1876–77 expedition to the Maclay-Coast (the North-east coast of Papua New Guinea) he conducted head-measurements on 148 living individuals of both sexes and described 23 Papuans crania [42]. He always emphasized that physical anthropology (the race anthropology) would remain merely an “unedifying doctrine” [*unerquickliches Studium*] without detailed field studies and profound anatomical investigations “on the section table” [42]. All measurements and observations were combined by Maclay with linguistic and ethnographic studies, leading to a general textual and visual description of a population.

The general result of all these efforts were exact anthropological and ethnographic portraits of “Melanesians”, “Polynesians” and “Australians”.

On a higher theoretical level, Maclay believed that racial differences are due to adaptation to local environments. For him, acclimatization of “the white race” in the Pacific region demonstrated that “whites” would be able to survive in the tropics only by crossing with indigenous people. He was convinced that in the future, the Pacific region would be populated by a “mix-race”, and that this would not necessarily be with a significant European component [45].

Maclay’s conclusions concerning the intellectual abilities of Melanesians were a result of thorough observations as well. In a lecture given in 1886 in St. Petersburg, and published in the same year, he explicitly spoke against the prejudices of the infamous French theorist of racial inequality Joseph Arthur de Gobineau (1816–1882) and specifically against his claim “L’homme est l’animal méchant par excellence” (Man is the wicked animal *par excellence*) [10, 45]. Maclay recalled, that when he began understanding the language of the Papuans and their relationships, he was surprised by their “gentleman-like” attitudes, democratic social structure and friendly family relations [45]. Maclay appealed to his ethnological collection at The Russian (St. Petersburg) Academy of Sciences, the missioner’s schools in various islands of Melanesia, the ability of Melanesians to learn mathematics (by contrast to Haeckel who questioned their “arithmetic abilities”) and other evidence. All were arguments against racial prejudices. Proceeding from his observations Maclay completely negated the idea of intellectual racial inequality [3]. This concept prompted him to fight for the rights of indigenous populations of Melanesia and generally for the rights of “dark-skinned” races. In the 1880s Maclay tried to convert his scientific reputation into political action. Unfortunately, his attempts failed and the northeastern part of the island became a German colony already in 1884, followed by the other territories.

Maclay avoided great theoretical generalizations and his exact position towards Darwinian evolutionary mechanisms remains enigmatic [68]. With all probability, he accepted some of Haeckel’s and Darwin’s Lamarckism, as he believed in the ability of “tribes” to quickly adapt to their local environments. He certainly did not believe in the Haeckelian concept of evolutionary progress as applied to extant human races. Maclay’s “tribes” and “races” differentiate under external influences but this does not make them “higher” or “lower” on the evolutionary tree, as in Haeckel’s case.

## Conclusions

**Fig. 16:**
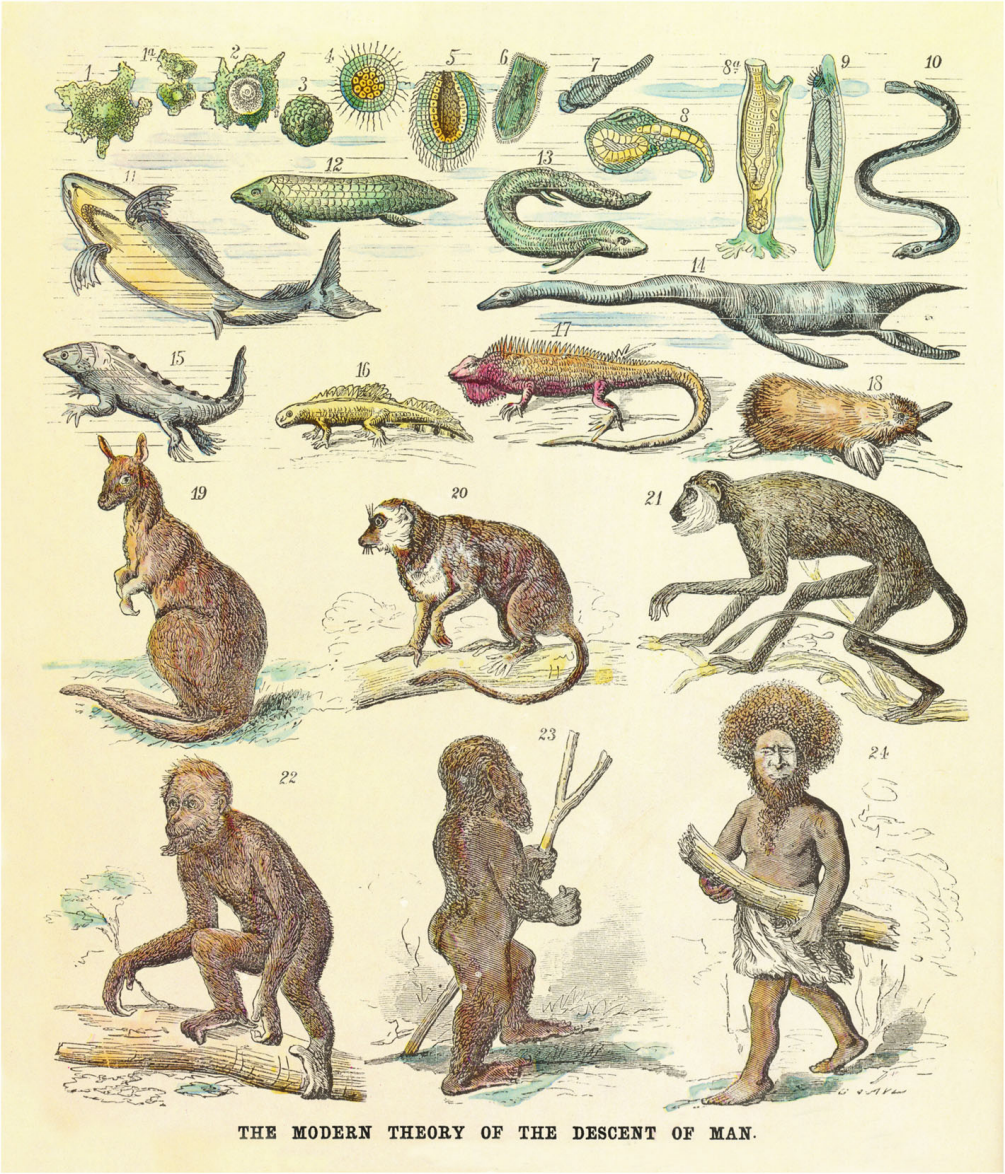
A visualization of Haeckel’s “The History of Creation” by G. Avery illustrating biological evolution from monera (1) to the Papuans (24), which arose from the primaeval man and represent “the oldest of all still living human species” (Scientific American, March 11, 1876, p. 167)

Haeckel believed in evolutionary progress and applied this belief to the evolution of human species as well (Fig. 16). Humans, according to him, evolved from a common ancestor *Pithecanthropus* and passed the stage of the “primordial man” *Homo primigenius (Protanthropi)*, which evolved on the “primordial continent” *Lemuria*. The Lemuria-man gave rise to the currently existing human species of which the Papuans belong to the most primitive and least advanced from *Protanthropi*. In accordance with the concept of natural selection and social Darwinian conceptual constructions, the Papuans were for Haeckel doomed to die out, being replaced by more progressive human races such as the Mediterranean. Haeckel applied to the evolution of human species the same principles as he applied to the evolution of sponges or apes. He proceeded from the assumption that every species that exists or ever existed on Earth evolved from a lower form by means of natural selection. It was for him a universal law. Looking for proof of this law in all areas of biology and anthropology Haeckel described species variation and reconstructed their hypothetical phylogenies [22, 40]. Human phylogeny, in his view, would necessarily reinforce this idea as currently, existing human species seem to be very distinct morphologically and culturally. In this way, Haeckel came to the sound idea that various human species could co-exist on Earth in the past and to the erroneous concept that they still populate the Earth. As we know now the very idea that Earth could be populated by various human species is not unscientific as, indeed, for example, *Homo sapiens*, *Homo Neanderthalensis* and *Denisova hominins* co-existed for a quite significant period [31, 53, 60].

As such the Papuans were for Haeckel a remnant of an ancient human form that survived in the New Guinea due to its isolation from the rest of the world, and its proximity to *Lemuria* (Fig. 15).

Maclay was Haeckel’s student and (for a certain period) his junior friend. He internalized the Haeckelian-Darwinian method of looking for geographic variation and applied it successfully in his zoological works of the Jena period. He learned from Haeckel that the best way to study living organisms is to observe them in their natural habitats in connection with their immediate living and inert environments. He traveled to New Guinea and other tropic regions to apply the same method to study humans and to empirically study the races of Polynesia, Melanesia, and other related regions, where Haeckel’s hypothetical primitive species could be found. In the course of his long-term stationary field studies, Maclay discovered that the Haeckelian view (and that of other theoreticians such as Fritz Müller) of the Papuans as a lower human species was erroneous on empirical grounds. But he proved Haeckel wrong by essentially using the method he learned in Jena with Haeckel. Although Maclay experienced many influences from other first rank researchers of his time, such as Karl Ernst von Baer, Alfred Russel Wallace, Rudolf Virchow, Carl Gegenbaur, etc., it was Haeckel, who initially taught him the Darwinian method and the subtleties of empirical research. This is an argument in favor of the idea that, although definitely racist from the contemporary viewpoint, and even from the viewpoint of many of anthropologists of those times, the Haeckelian racial scheme appeared as a result of lacking in empirical anthropological data rather than as a necessary outcome of the method he relied on. As Haeckel’s student, Maclay shows us what happens when that method was applied to a richer field of anthropological observations.

Unfortunately, Maclay’s observations had little influence in the Western world, and could not prevent growing racial thinking in Europe and especially in Germany in the first half of the twentieth century. In their infamous book *Menschliche Erblehre und Rassenhygiene (Eugenik)* [Human Heredity and the Racial Hygiene (Eugenics)] first printed in 1921 and republished altogether five times, Erwin Baur, Eugen Fischer, and Fritz Lenz appealed again to the “hair-argument” to prove the lower value of “Papua-Melanesians”; their ulotrichan hair [Kraushaarigkeit] served the authors as an indicator of Papuan retardation [2].

By contrast, Maclay’s exact observations contributed to the growing scientific anti-racism, which became a commonly accepted position nowadays [7].

## Data Availability

Yes

## Footnotes

1 Leo Tolstoy to P.I. Biryukov, on May 24th 1908.

2 Leo Tolstoy to Miklucho-Maclay on September 25th, 1886.
